# Traumatic Versus Atraumatic Causes of Shoulder Impingement Syndrome: A Systematic Review of Pathophysiology and Outcomes

**DOI:** 10.7759/cureus.93628

**Published:** 2025-10-01

**Authors:** Muhammad Irfan Akram, Kunjan Yogesh Barot, Rao Junaid Saleem, Abdullah Elrefae, Hassan Imtiaz, Kshitij Srivastava, Mohammad Shishtawi, Safeer Ahmad Javid, Muhammad Rizwan Umer, Shahzaib Ahmad

**Affiliations:** 1 Trauma and Orthopedics, Ipswich Hospital, Ispwich, GBR; 2 Trauma and Orthopedics, Poole General Hospital, Poole, GBR; 3 General and Colorectal Surgery, Northwick Park Hospital, London, GBR; 4 Trauma and Orthopedics, AlBashir Hospital, Amman, JOR; 5 Trauma and Orthopedics, University Hospitals Dorset NHS Foundation Trust, Poole, GBR; 6 Trauma and Orthopedics, Northwick Park Hospital, London, GBR; 7 General Surgery, Princess of Wales Hospital-Cwm Taf Morgannwg University Health Board, Bridgend, GBR; 8 Trauma, Royal Sussex County Hospital, Brighton and Hove, GBR; 9 Trauma and Orthopedics, Royal Sussex County Hospital, Brighton and Hove, GBR; 10 General Surgery, Civil Hospital Karachi, Karachi, PAK

**Keywords:** atraumatic, outcomes, pathophysiology, shoulder impingement syndrome, systematic review, traumatic

## Abstract

Shoulder impingement syndrome (SIS) is a common musculoskeletal disorder caused by traumatic or atraumatic factors, resulting in pain, functional limitation, and reduced quality of life. This systematic review aimed to summarize the pathophysiology, anatomical changes, and functional outcomes of traumatic versus atraumatic SIS. A comprehensive search of PubMed, Embase, Scopus, and the Cochrane Library up to 2025 was conducted following Preferred Reporting Items for Systematic Reviews and Meta-Analyses (PRISMA) 2020 guidelines, including studies reporting clinical outcomes and pathophysiological findings in humans. Five studies comprising 467 participants were included. Traumatic SIS, often following acute injuries or post-acromioclavicular (AC) joint fixation, was associated with rotator cuff tears, labral lesions, and anatomical changes, leading to pain, limited range of motion, and delayed recovery. Atraumatic SIS, common in overhead athletes, resulted from repetitive microtrauma, causing tendon degeneration, subacromial narrowing, and deficits in strength, motion, and dynamic balance. Both etiologies shared rotator cuff and bursal inflammation. Traumatic SIS frequently requires surgical intervention, whereas atraumatic SIS is managed conservatively with structured rehabilitation. Differentiating etiologies is essential to optimize outcomes, prevent recurrence, and guide treatment strategies, and further long-term studies are warranted to evaluate functional recovery and intervention effectiveness.

## Introduction and background

Shoulder impingement syndrome (SIS) is a common musculoskeletal disorder characterized by pain, restricted movement, and functional limitations [1]. SIS typically arises from compression of the rotator cuff and subacromial bursa against the anterolateral acromion, causing anterolateral shoulder pain that worsens at night and with overhead activity. Atypical cases may result from factors like os acromiale, subcoracoid issues, acromioclavicular (AC) joint hypertrophy, rotator cuff weakness, or scapular dyskinesis. Diagnosis relies on history, examination, and imaging, and most cases respond to nonsurgical treatment, with surgery reserved for persistent structural causes. Although SIS can affect anyone, it is more frequently seen in athletes involved in repetitive overhead activities, manual laborers, and individuals with structural variations in the subacromial space. The syndrome can considerably affect quality of life by limiting daily activities and athletic performance, and if left untreated, it may progress to chronic tendon degeneration [2].

Understanding the underlying mechanisms and contributing factors of SIS is crucial for effective prevention and management. Traumatic SIS typically results from acute injuries, such as falls, direct shoulder trauma, or AC joint dislocations. These events can cause immediate damage to the rotator cuff, labrum, or bursa, triggering inflammation, pain, and mechanical dysfunction [3]. In some cases, surgical interventions, including tendon repair or hook plate fixation, are necessary to restore stability and prevent persistent impingement. Post-injury or post-surgical anatomical changes can further predispose patients to recurrent episodes of SIS, highlighting the importance of precise surgical technique and structured rehabilitation [4]. In contrast, atraumatic SIS develops gradually due to repetitive microtrauma and overuse, particularly in athletes who frequently perform overhead movements, such as swimmers or baseball pitchers.

Chronic overloading can lead to tendon degeneration, narrowing of the subacromial space, and impaired dynamic stability of the shoulder. Over time, these changes compromise shoulder function and increase the risk of tendon tears. Management generally emphasizes conservative approaches, including physical therapy, strengthening exercises, and activity modification, although persistent cases may require surgical decompression. Despite differing origins, traumatic and atraumatic SIS share similar pathological features, including tendon inflammation, bursal irritation, and subacromial compression. Distinguishing between these etiologies is essential for clinicians to determine appropriate treatment strategies, rehabilitation plans, and prognostic expectations. This systematic review aims to consolidate current evidence regarding the pathophysiology, anatomical implications, and clinical outcomes of traumatic versus atraumatic SIS, offering a comprehensive resource to guide clinical decision-making and future research.

## Review

Materials and methods

Search Strategy

A comprehensive literature search was conducted in accordance with Preferred Reporting Items for Systematic Reviews and Meta-Analyses (PRISMA) 2020 guidelines [5]. Databases including PubMed, Embase, Scopus, and the Cochrane Library were used to identify studies on traumatic and atraumatic SIS published up to 2025. Search terms included “shoulder impingement,” “traumatic,” “atraumatic,” “overuse,” “pathophysiology,” and “outcomes.” Boolean operators were used to refine the search. References of relevant articles were manually screened to ensure comprehensive coverage of eligible studies.

Eligibility Criteria

The eligibility criteria for this systematic review were structured using the PICO framework [6]. Population (P) included human participants diagnosed with SIS. Intervention/Exposure (I) was traumatic or atraumatic causes of SIS, while Comparator (C) included either healthy controls, non-SIS participants, or alternative management strategies depending on study design. Outcomes (O) focused on clinical measures such as range of motion, strength, functional recovery, recurrence rates, and pathophysiological or anatomical findings. Studies were included if they clearly specified the etiology of SIS and reported relevant outcomes. Exclusion criteria were case reports, animal studies, editorials, and conference abstracts to maintain methodological quality and relevance. Only peer-reviewed publications were considered to ensure reliability and scientific rigor.

Study Selection

All identified studies were independently screened by two reviewers. Titles and abstracts were first assessed for relevance, followed by full-text review for studies meeting initial eligibility. Any discrepancies were resolved through discussion or consultation with a third reviewer to ensure objectivity and consensus. Studies fulfilling all inclusion criteria were included in the final review for data extraction.

Data Extraction

Data were independently extracted from the final set of included studies using a standardized extraction sheet. Data were systematically extracted by two independent reviewers using a standardized form to minimize bias. Extracted information included study characteristics, sample size, exposure type, comparator groups, clinical outcomes, pathophysiological findings, and anatomical impact. This approach ensured a consistent and comprehensive collection of relevant data across all included studies.

Risk of Bias Assessment

The risk of bias was assessed using validated tools according to the study design. Cohort studies were evaluated with the Newcastle-Ottawa Scale [7], case series with the Joanna Briggs Institute checklist [8], and systematic reviews using A Measurement Tool to Assess Systematic Reviews (AMSTAR 2) [9]. Each study was rated as low, moderate, or high risk of bias, with justifications provided based on study design, sample size, data collection methods, and methodological rigor.

Data Synthesis

Due to heterogeneity in study design, populations, and outcome measures, data were synthesized narratively rather than quantitatively. The synthesis focused on summarizing the pathophysiology, anatomical changes, and functional outcomes associated with traumatic versus atraumatic SIS, highlighting differences that inform clinical decision-making and management strategies.

Results

Study Selection Process

**Figure 1:**
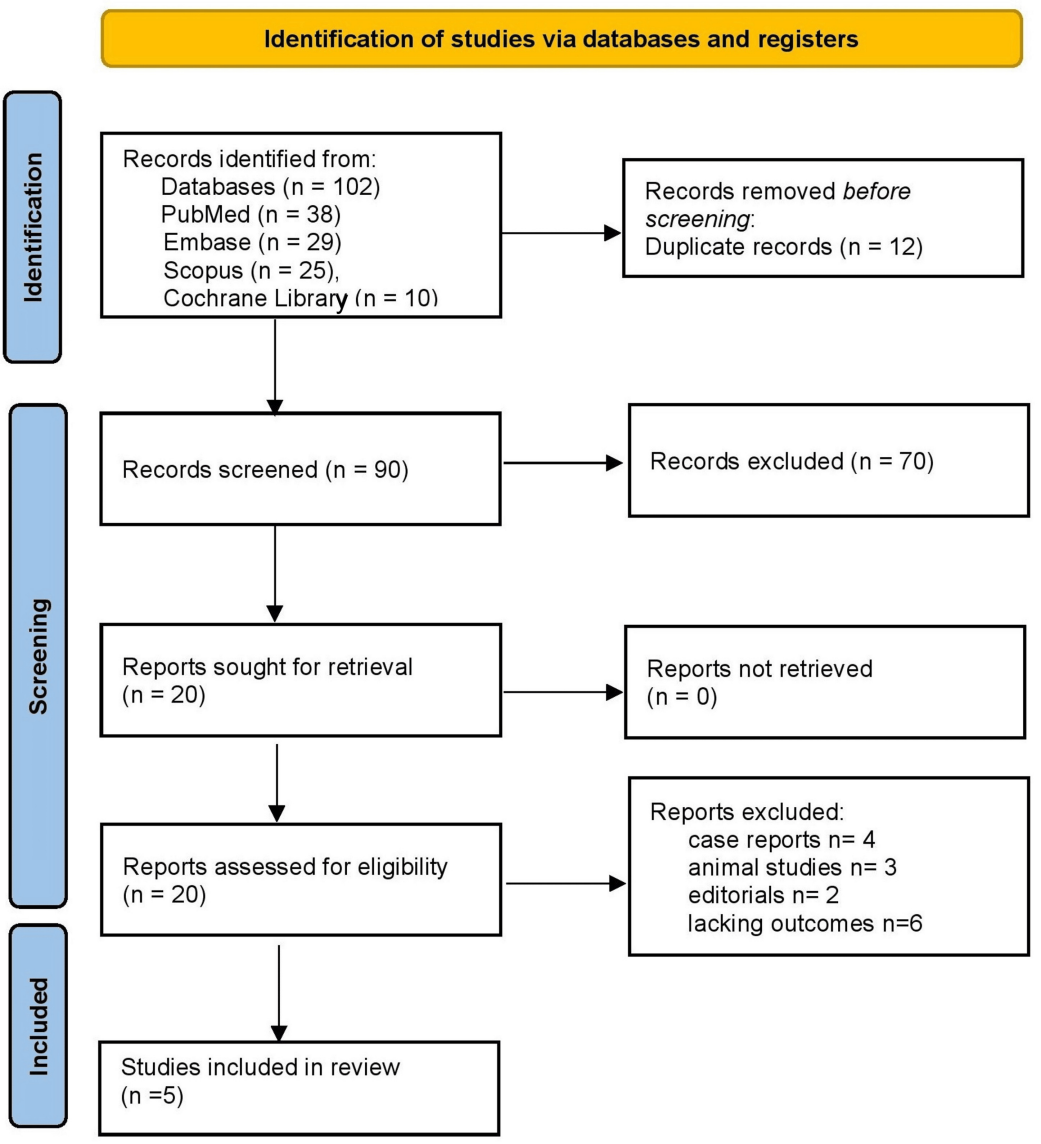
Preferred Reporting Items for Systematic Reviews and Meta-Analyses (PRISMA) guideline 2020.

Figure 1 demonstrates the study selection process following the PRISMA guidelines. A total of 102 records were identified from four electronic databases: PubMed (n = 38), Embase (n = 29), Scopus (n = 25), and the Cochrane Library (n = 10). After removing 12 duplicate records, 90 articles were screened based on titles and abstracts for relevance. Of these, 70 records were excluded as they did not meet the inclusion criteria, leaving 20 articles for full-text assessment. Full-text review revealed that 15 articles did not meet eligibility criteria, including four case reports, three animal studies, two editorials, and six conference abstracts. No articles were unavailable for retrieval. After applying these exclusions, a total of five studies were included in the systematic review.

Characteristics of the Selected Studies

Table 1 summarizes the characteristics of the five studies included in this systematic review. Ding Xu et al. (2021) examined 312 patients who underwent hook plate fixation for AC joint dislocation, reporting a 27.8% incidence of traumatic SIS, with pathophysiology linked to mechanical factors from surgical fixation and anatomical alterations in acromion and hook plate positioning [10]. Youngwook Kim et al. (2020) evaluated 58 National Collegiate Athletic Association (NCAA) Division I overhead athletes with a history of atraumatic SIS, demonstrating that repetitive overhead activity-related microtrauma resulted in reduced shoulder range of motion, strength, and impaired dynamic balance compared to uninjured athletes [11]. Myoung Gi On et al. (2019) studied 14 elite athletes from the 2018 Winter Olympics who sustained traumatic SIS due to acute injuries, reporting acute inflammation, contusions, rotator cuff tears, and labral lesions that affected recovery time [12]. Véronique Lowry et al. (2024) conducted a systematic review of 26 clinical practice guidelines, highlighting evidence-based management strategies for both traumatic and atraumatic SIS while noting variability in recommendations for surgical versus conservative interventions [13]. Finally, Wilk et al. (2009) investigated 83 professional baseball players with atraumatic SIS due to overuse, demonstrating tendon degeneration, subacromial space narrowing, and overuse-related impingement, emphasizing the importance of preventive measures and rehabilitation in overhead athletes [14]. Collectively, these studies provide comprehensive insight into the etiology, pathophysiology, anatomical impact, and clinical outcomes of traumatic and atraumatic SIS, supporting the need for tailored management strategies.

**Table 1:** Characteristics of the selected studies. SIS: shoulder impingement syndrome; AC: acromioclavicular; CPG: clinical practice guideline; ROM: range of motion; NCCA: National Collegiate Athletic Association

Authors & Year	Population (P) / Sample Size	Exposure / Condition (I)	Comparator (C)	Outcomes (O)	Pathophysiological Findings	Anatomical Impact
Ding Xu et al., 2021 [10]	312 patients with AC joint dislocation	Traumatic SIS post-hook plate fixation	Non-SIS group	Incidence of SIS, risk factors	Surgical fixation-related mechanical factors	SIS incidence: 27.8%; anatomical changes in acromion and hook plate positioning
Youngwook Kim et al., 2020 [11]	58 NCAA Division I overhead athletes	Atraumatic SIS history vs. uninjured controls	Uninjured athletes	Shoulder range of motion, strength, and dynamic balance	Overhead activity-related microtrauma	Reduced range of motion and strength in previously injured athletes
Myoung Gi On et al., 2019 [12]	14 elite athletes from the 2018 Winter Olympics	Traumatic SIS due to acute injury	Non-traumatic shoulder pain	Injury type, recovery time	Acute trauma-related inflammation	Contusions, rotator cuff injuries, and labral tears
Véronique Lowry et al., 2024 [13]	Systematic review of 26 CPGs	Traumatic and atraumatic SIS management guidelines	N/A	Guideline quality, treatment recommendations	Evidence-based management strategies	Variability in surgical vs. conservative recommendations
Wilk et al., 2009 [14]	Overhead athletes (general), 83 professional baseball players	Atraumatic SIS due to overuse	N/A	Injury prevention, rehabilitation strategies	Overuse leading to tendonitis and impingement	Tendon degeneration, subacromial space narrowing

Risk of Bias Assessment

Table 2 summarizes the risk of bias for the included studies, which was assessed using validated tools appropriate to each study design. Ding Xu et al. (2021), a retrospective cohort, was rated moderate using the NOS due to its retrospective nature, although outcome reporting was consistent and inclusion criteria were clear [10]. Kim et al. (2020), a case-control study, was rated low on NOS for its clear participant selection, blinded assessment, and robust outcome measures [11]. Myoung Gi On et al. (2019), a case series, was rated moderate using the JBI checklist due to a small sample size but detailed clinical and pathophysiological data [12]. Both Véronique Lowry et al. (2024) and Wilk et al. (2009) reviews were assessed with AMSTAR 2 and rated low risk, reflecting comprehensive search strategies, transparent methodology, and clear evidence synthesis [13,14].

**Table 2:** Risk of bias assessment. NOS: Newcastle-Ottawa Scale; JBI: Joanna Briggs Institute Checklist; AMSTAR 2: A Measurement Tool to Assess Systematic Reviews

Study	Study Design	Risk of Bias Tool	Risk of Bias Rating	Justification
Ding Xu et al., 2021 [10]	Retrospective cohort	Newcastle-Ottawa Scale (NOS)	Moderate	Retrospective design, but consistent outcome reporting and clear inclusion criteria
Youngwook Kim et al., 2020 [11]	Case-control	Newcastle-Ottawa Scale (NOS)	Low	Clear participant selection, blinded assessment, and robust outcome measures
Myoung Gi On et al., 2019 [12]	Case series	Joanna Briggs Institute (JBI) Checklist	Moderate	Small sample size, but detailed clinical and pathophysiological data
Véronique Lowry et al., 2024 [13]	Systematic review	AMSTAR 2	Low	Comprehensive search, transparent methodology, clear synthesis of evidence
Wilk et al., 2009 [14]	Review	AMSTAR 2	Low	Established methodology, clear outcome reporting, widely cited evidence

Discussion

SIS is a heterogeneous musculoskeletal disorder with distinct traumatic and atraumatic etiologies, yet both share overlapping pathological mechanisms. Traumatic SIS typically arises from acute injuries such as falls, direct shoulder trauma, or AC joint dislocations, resulting in immediate damage to the rotator cuff, labrum, and subacromial bursa. Ding Xu et al. (2021) reported a 27.8% incidence of traumatic SIS in 312 patients following hook plate fixation for AC joint dislocations. They demonstrated that mechanical factors related to surgical fixation and anatomical alterations in acromion and hook plate positioning significantly contributed to post-surgical impingement, with outcomes including persistent pain, limited range of motion, and delayed return to daily activities [10]. Acute trauma triggers inflammatory cascades, including cytokine release and vascular congestion, leading to edema, pain, and functional impairment. Postoperative anatomical changes can further prolong recovery and increase the risk of recurrent SIS, underscoring the importance of precise surgical technique and structured rehabilitation to restore optimal shoulder mechanics [15].

Atraumatic SIS develops gradually due to repetitive microtrauma and overuse, predominantly in overhead athletes such as baseball pitchers, swimmers, and volleyball players. Youngwook Kim et al. (2020) evaluated 58 NCAA Division I overhead athletes with a history of atraumatic SIS and found significant reductions in shoulder range of motion, strength, and upper-quarter dynamic balance compared to uninjured controls. These functional deficits translated to impaired performance, increased injury risk, and longer recovery periods following minor shoulder stress [11]. Wilk et al. (2009) studied 83 professional baseball players with overuse-related SIS and reported tendon degeneration, subacromial space narrowing, and chronic impingement, emphasizing the need for preventive strategies, targeted rehabilitation, and monitoring of functional outcomes such as throwing velocity, endurance, and shoulder stability [14].

Traumatic SIS in elite athletes also leads to significant short-term functional limitations. Myoung Gi On et al. (2019) evaluated 14 Winter Olympic athletes who sustained traumatic SIS and observed acute inflammation, contusions, rotator cuff injuries, and labral tears. Outcomes included prolonged recovery times, limitations in overhead activity, and the necessity for individualized rehabilitation programs to regain pre-injury performance levels. Lowry et al. (2024), in a systematic review of 26 clinical practice guidelines, highlighted evidence-based management strategies for both traumatic and atraumatic SIS. The review underscored variability in surgical versus conservative recommendations and emphasized that functional outcomes, including pain reduction, range of motion restoration, and return to sport or work, are heavily influenced by early diagnosis and tailored management [12].

Despite differences in etiology, both traumatic and atraumatic SIS share common pathological features, including rotator cuff and subacromial bursa inflammation, altered scapulohumeral kinematics, and secondary muscular adaptations. Functional outcomes are determined by etiology, severity of structural damage, and adherence to rehabilitation protocols. Traumatic cases may require surgical correction to restore anatomical alignment and prevent recurrent impingement, while atraumatic cases primarily benefit from conservative management strategies such as physical therapy, activity modification, and scapular stabilization. Early recognition, accurate differentiation of etiology, and individualized rehabilitation plans are crucial to optimize recovery, improve functional outcomes, reduce recurrence, and enhance quality of life. This review is limited by the small number of included studies and heterogeneity in study designs, populations, and outcome measures. Additionally, long-term follow-up data on functional recovery, recurrence, and comparative effectiveness of surgical versus conservative management remain limited, highlighting the need for future prospective studies with standardized outcome assessment.

## Conclusions

Traumatic and atraumatic SIS exhibit distinct etiologies but share overlapping pathological mechanisms, including rotator cuff and bursal inflammation, altered shoulder kinematics, and functional impairment. Traumatic SIS, often resulting from acute injuries or post-surgical anatomical changes, is associated with abrupt structural damage, prolonged recovery, and a higher risk of recurrence, frequently requiring surgical intervention. In contrast, atraumatic SIS develops gradually due to repetitive microtrauma and overuse, leading to tendon degeneration, subacromial space narrowing, and functional deficits, with conservative management and structured rehabilitation being the mainstay of treatment. Differentiating between these etiologies is essential for tailoring management strategies, optimizing functional outcomes, and preventing long-term complications. Future research should focus on long-term comparative studies evaluating functional recovery, recurrence rates, and the effectiveness of surgical versus conservative interventions.
